# Early Childhood Dietary Exposure of Aflatoxins and Fumonisins in Tanzania: Role in Child Stunting and Possible Interventions (2003–2024)

**DOI:** 10.1002/fsn3.71346

**Published:** 2025-12-22

**Authors:** Clara Mollay

**Affiliations:** ^1^ School of Life Sciences and Bioengineering Nelson Mandela African Institution of Science and Technology (NM‐AIST) Arusha Tanzania

**Keywords:** aflatoxin exposure, childhood stunting, complementary foods, dietary exposure to mycotoxin, fumonisins exposure, growth impairment

## Abstract

One of the major sources of public health concern in many developing countries is child stunting, which accounts for more than a third of all deaths of children below the age of 5 years worldwide. Although child stunting generally seems to be on a downward trend in the country, the present prevalence is still very high based on international standards. Several studies have established that the extent of early childhood dietary exposure to aflatoxins (AFs) and fumonisins (FBs) in the country occurs through complementary feeding. However, there is a paucity of information on whether this is linked to child stunting. This review discusses child stunting and nutrition and strives to draw a link between early childhood dietary exposure to AFs and FBs and child stunting in Tanzania. The review further outlines the mechanisms of mycotoxin‐induced growth impairment and deliberates on the past, present, and future interventions that can be useful in alleviating the problem of stunting that is linked to these mycotoxins. Such information can be crucial for developing and adopting appropriate interventions that can contribute to the further reduction of child stunting in Tanzania.

## Graphical abstract

Childhood stunting is a major public health concern, and dietary exposure to aflatoxins and fumonisins during early life is associated with child stunting in Tanzania. The causal link between aflatoxin and/or fumonisin exposure and child stunting has been less well explained. Addressing mycotoxin‐induced growth impairment requires a comprehensive approach that considers past, present, and future interventions by multiple stakeholders.

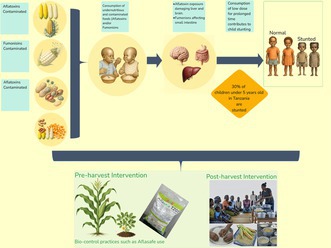

## 1 Introduction

According to the Food and Agriculture Organization (FAO 2023), globally, over one in five children (148.1 million) under 5 years of age were stunted in 2022. In Tanzania, over 2.8 million children are stunted. Stunting is a multifactorial growth impairment due to undernutrition, recurrent infections, substandard health care, or environmental enteropathy (Nandy and Miranda 2008; Smith et al. 2015). Stunting may negatively affect both a child's mental and physical development (Black et al. 2013; UNICEF et al. 2012), increase vulnerability to infections (URT and UNICEF 2016), as well as severity and delayed recovery from infections.

Childhood stunting affects about one‐third of children below 5 years of age and accounts for about 15% of children under five mortalities globally (Black et al. 2013; Danaei et al. 2016). However, developing countries continue to bear the biggest brunt of this burden (UNICEF et al. 2017). For instance, about 32.5% of children in developing countries are stunted as they do not reach the standard heights for their ages (de Onis et al. 2012). In addition to having the greatest estimated occurrence of stunting, the African region also has the least rate of improvement. In Africa, the prevalence of stunting among children in the same age category is 30%, which is significantly higher than the global estimate of 22.3% (FAO 2023). According to Safari et al. (2013) and Kulwa et al. (2015), stunting is a major health concern among Tanzanian infants and young children (IYC). Although the rate of stunting among children below 5 years of age in Tanzania decreased from 42% in 2010 (NBS and ICF Macro 2011) to 30% according to the recent Tanzania Demographic and Health Survey and Malaria Indicator Survey (TDHS 2022), stunting is still a great cause of public health concern in the country.

While child stunting has previously mostly been associated with undernutrition, infectious diseases, and sanitary/hygienic factors in different regions of the world (Banerjee and Dwivedi 2020; Katona and Katona‐Apte 2008; Millward 2017; Torlesse et al. 2016), greater concern has emerged in the last two decades regarding the link between the dietary exposure of children to mycotoxins and impaired growth. In particular, new studies have concentrated on the role of fumonisins (FBs) and aflatoxins (AFs) in common complementary diets in the growth impairment of children (Anitha et al. 2020; G. Chen, Gong, et al. 2018; Rasheed et al. 2021; Turner 2013; Wild and Gong 2010). While FBs are primarily produced by *Fusarium* spp., AFs are metabolic products of *Aspergillus* fungi, which are common contaminants of food crops used as ingredients for complementary foods (CFs) in tropical, subtropical, and temperate climates (Kimanya et al. 2014). Despite the growing link between exposure to mycotoxins and growth impairment, the biological mechanisms of growth impairment are still unclear (Perdoncini et al. 2020; Söylemez et al. 2020). This review describes child nutrition and stunting in Tanzania and strives to draw a link between early childhood dietary exposure to AFs and FBs and child stunting in the country. The review further outlines the mechanisms of growth impairment and deliberates on the past, present, and future interventions that can be useful for alleviating the problem of stunting that is linked to these mycotoxins. Such information can be critical in developing and adopting appropriate interventions that can reduce child stunting in Tanzania. Box 1 highlights the main messages from this review.

BOX 1 **Key messages.**

- Childhood stunting is a major public health concern in Tanzania.
- Exposure to aflatoxins and fumonisins during early life is associated with child stunting in Tanzania.
- The causal link between aflatoxin and/or fumonisin exposure and child stunting has been less well explained.
- Addressing mycotoxin‐induced growth impairment requires a comprehensive approach that considers past, present, and future interventions by multiple stakeholders.

## 2 Prevalence of Stunting in Children (6–59 Months) in Tanzania

The stunting of young children and infants is a major health problem in Tanzania (Kulwa et al. 2015; Safari et al. 2013). On the basis of the recent TDHS in the country, approximately 30% of young children (below the age of 5 years) are stunted, while 9% are severely stunted, which places the country among the 10 most‐affected countries globally. Nevertheless, this is a huge decrease from the prevalence of 44% in 2004–2005 (TDHS 2022). However much this decline seems over two decades, the present prevalence of stunting in the country remains one of the highest globally as per the World Health Organization (WHO 2019) classifications, with Tanzania still among the 14 countries that share 80% of the global burden of stunting.

Like many low‐income countries, stunting is widespread in Tanzania. However, there also exists a significant regional variation of child stunting in the country (TDHS 2022). According to this report, the Iringa region was leading with high stunting prevalence compared to all other regions in the country.

Apart from environmental factors, studies have also revealed that child stunting in Tanzania could be subject to the prevailing socioeconomic factors (Chirande et al. 2015; Khamis et al. 2019; Mgongo et al. 2017). For instance, urban‐dwelling children are generally less susceptible to stunting compared to their counterparts in rural areas (Kejo et al. 2018; Makori et al. 2018; Mrema et al. 2021; Sunguya et al. 2019). This was recently confirmed in the 2022 TDHS where the stunting rates of children in rural Tanzania were above average compared to the urban areas (TDHS 2022). For instance, the stunting rates were 20.5% and 33.4% in urban and rural areas, respectively (TDHS 2022). Generally, rural areas account for the highest number of stunted children (Mamiro et al. 2005; NBS and ICF Macro 2011; Shirima et al. 2015). This may be due to mothers in rural areas tending to spend more time on their farms and having less time to feed their babies (Makori et al. 2018). However, the prevalence of stunting in rural settings may be reduced during postharvest when more food is available for households (Muhimbula et al. 2019).

Stunting is associated with age, indicating that the risk of exposure to AFs increases with age. Older children (12–24 months) in Tanzania are seemingly more exposed to stunting compared to their younger counterparts (6–11 months) (Altare et al. 2016; Chirande et al. 2015; Makori et al. 2018). According to Victora et al. (2010), stunting rates begin to increase approximately 3 months after birth and then reduce at about the age of 2 years. The risk of child stunting rises at 7–24 months, declines at 25–36 months, and then rises again at 37–59 months. It is during the first 2 years of life that children generally accrue growth delays, with stunting climaxing at about 2–3 years, after which they stabilize (Nyaruhucha et al. 2006). The peak age (24–35 months) is an important nutritional milestone when a child has stopped breastfeeding, is mostly independent, and has already been introduced to complementary feeding in the form of regular family foods (Sunguya et al. 2019). The high prevalence of stunting observed beyond 0–23 months may thus be associated with improper food supplementation during weaning or complementary feeding (Mittal et al. 2007). In the analysis done by Sunguya et al. (2019), 24–35‐month‐old children were more exposed to stunting compared to those aged 0–11 months. The reduced level of stunting in children less than 7 months old could be attributed to the benefits of exclusively breastfeeding children in the first few months of life (NBS and ICF Macro 2011). Similarly, the higher risk of stunting at 7–24 months could also be attributed to the challenges of the introduction of CFs (Mbwana et al. 2017). This is discussed in Section 3.

Stunting has also been associated with sex. For instance, 42.6% of male children under 5 years of age were stunted compared to only 41.2% of female children in the same age bracket in six districts in the Kilimanjaro region (Mgongo et al. 2017). Similar results have previously been reported by Altare et al. (2016) while investigating the factors associated with stunting among pre‐school children aged 6–59 months in the Southern Highlands of Tanzania, where the results showed that male children had higher odds of being stunted than female children. According to Chirande et al. (2015), male children aged 0–59 months were found to have a higher risk of being stunted or severely stunted compared to females. This finding is consistent with a finding reported from a meta‐analysis of 16 demographic and health surveys of 10 countries in sub‐Saharan Africa, in which male children were found to be consistently more likely to become stunted compared to their female counterparts (Wamani et al. 2007). The same results have also been recorded nationally with average stunting rates of 33.3% and 26.2% for male and female children under 5 years of age, respectively (TDHS 2022). According to Mrema et al. (2021), who also reported similar results in the high and lowland areas of Kilosa District, this could be due to cultural beliefs that boys have a higher appetite and low satiety compared to girls. The gender differences could also be due to behavioral patterns employed by girls, such as spending more time with their mothers in the kitchen, compared to boys who are hyperactive and spend most of their time playing away from their homes where they can skip several meals.

## 3 Early Childhood Diets in Tanzania

Like the rest of the world, IYC in Tanzania are often introduced to CFs during complementary feeding when breast milk is no longer adequate for their nutritional needs (Burdette et al. 2006; WHO/PAHO 2003). Despite breastfeeding being able to prolong beyond the second year of life, the target age for complementary feeding is normally 6–24 months (Dewey and Adu‐Afarwuah 2008). Contrary to the recommendation of the WHO, 16%, 41%, and 73% of infants < 2 months of age, between 2 and 3 months, and between 4 and 5 months, respectively, were already introduced to CFs in the 2015–2016 TDHS (2016). According to Shirima et al. (2001), the introduction of solid CFs to infants in the country begins at the ages of 1–8 and 2–6 months in the rural and urban areas, respectively. However, other studies have established that even children as young as 2 months old are introduced to porridge made of maize ( *Zea mays* ) and sometimes supplemented with milk (Kulwa et al. 2015; NBS and ICF Macro 2011; Nyaruhucha et al. 2006). In a survey by Kulwa et al. (2006), the mean age for the introduction of cereal‐based CFs in urban areas was reported as 3 months, even in urban areas like Dar es Salaam. Information regarding exclusive breastfeeding and the introduction of CFs in Tanzania, as compiled during the 2010 TDHS, indicates that 11% of children below 2 months of age, 34% of children aged 2–3 months, and 65% of children aged 4–5 months are given CFs (NBS and ICF Macro 2011). Most infants are introduced to CFs before the age of 6 months, and the main food given to them is composed of maize in the form of thin porridge (*uji*) (Mamiro et al. 2005; Nyaruhucha et al. 2006).

According to a recent national survey, 19% of infants were introduced to soft, semi‐solid, or solid foods at an early age (0–1 year). This figure increases to 32% by 2–3 months and reaches 55% by 4–5 months (TDHS 2022), which contradicts the WHO's recommendation of exclusive breastfeeding for the first 6 months of life. Such recommendations are aimed at ensuring adequate nutrient provision, protecting against common childhood illnesses, and promoting long‐term health benefits. Approximately 81% of children aged 6–23 months did not achieve the minimum dietary diversity (MDD), while 67% reported a low meal frequency (TDHS 2022). Research conducted in Tanzania indicated that children who did not meet the MDD had a significantly higher likelihood of being stunted (AOR = 1.37, 95% CI; 1.13–1.65). Similar associations have been reported in other studies in Tanzania (Masuke et al. 2021; Shilugu and Sunguya 2019). Ongoing education for mothers, carers, and the community regarding appropriate complementary feeding practices, including the use of safe and nutritious diverse foods, could help reduce child stunting in Tanzania.

All through the transition from breastfeeding to complementary feeding, IYC are provided with small quantities of solid and semi‐solid foods during the day. Although the timely introduction of CFs promotes good nutrition and growth in IYC, the complementary feeding period is a vital window for growth retardation, nutritional deficiency, and an increased infection with contagious diseases (Mahgoub et al. 2006). Nutritional deficiencies are especially prevalent where children are predominantly fed on high levels of cereal‐based CFs (Bankole and Adebanjo 2003). Sub‐optimal feeding practices, frequent infections, poor quality of CFs, and micronutrient deficiencies contribute to the increased rates of malnutrition and mortality among IYC (Black et al. 2008). Feeding practices, including time of introduction and type and/or quality and quantity of CFs given, are some of the most significant factors affecting the nutritional status of a child (Kulwa et al. 2006).

Adequate nutrition during complementary feeding generally remains a big challenge (Dewey and Vitta 2013). For several years, the selection of CFs has been dictated by traditional and cultural practices, ignoring an appropriate focus on their nutritive values. Besides, the issue of food safety is rarely taken into consideration alongside the choices and/or preparation of CFs. A majority of Tanzanian households depend on composite home‐made cereal‐based CFs (Muhimbula and Issa‐Zacharia 2010) constituted of numerous cereals including millet ( *Pennisetum glaucum* ), maize, sorghum ( *Sorghum bicolor* ), finger millet (*Eleusine coracona*), and rice ( *Oryza sativa* ) as dictated by accessibility and cost (Mamiro et al. 2005). Due to their excellent energy content, cereals serve as the main ingredients in the preparation of CFs (Klerks et al. 2019). Maize, being the staple food in Tanzania, is used as the chief ingredient in the preparation of baby foods (Mamiro et al. 2005; Nyaruhucha et al. 2006). For instance, in central Tanzania, maize is the main ingredient of complementary porridge, with little or no inclusion of vegetables or protein‐rich products (Muhimbula and Issa‐Zacharia 2010). In rural Tanzania, it was previously shown that the infants' per‐capita consumption of maize as a single staple by infants was as high as 43 g/day, with little or no diversification (Kimanya et al. 2008). These largely cereal‐based infant diets not only deprive the infants of proteins and essential amino acids like tryptophan and lysine (Osundahunsi and Aworh 2003) but also other essential nutrients (Aron et al. 2017).

Although maize‐based gruels are the most common forms of CFs in Tanzania, sorghum, millet, rice, cassava, potatoes, and yams are also available (Mamiro et al. 2005). Other ingredients like sugar or salt and other cereal grains like rice, finger millet, and peanuts can be added, but differ between households (Muhimbula and Issa‐Zacharia 2010). Vegetables, fruits, legumes, meat, poultry, fish, eggs, roots and tubers, milk, and milk products are also used as CFs in Tanzania (NBS and ICF Macro 2011). Nevertheless, the combination and proportion of each ingredient are solely dependent on the mothers or caregivers. The CFs are mainly made into thin porridges of varying composition and consistency depending on a child's age. For instance, 3–5‐month‐old children tend to consume very thin maize porridge, whereas older ones (6–11 months) are fed on thicker porridge made from maize or composite flour.

Peanut‐enriched flour is sometimes employed as an infant‐weaning food in Tanzania owing to its high protein content (Kuhumba et al. 2018). The use of groundnuts as an ingredient of CFs is especially advocated for, not only because of their taste but also their availability and cultural acceptability as a source of fats and proteins (Lymo and Muzanila 2014). The common composite flours in most communities have a 70:30 cereal‐legume ratio to increase their protein contents (Dewey and Adu‐Afarwuah 2008). According to the United States Department of Agriculture (USDA 2015), peanuts are not only inexpensive but also a good source of protein (25.8 g of protein/100 g) and energy (567 kcal/100 g).

Despite the production and availability of different nutrient‐dense crops such as groundnuts, sorghum, pigeon pea, rice, and pearl millet, their inclusion in CFs generally remains on a low scale compared to maize (Kimanya et al. 2008). The limited inclusion of such nutritionally beneficial crops in the diets of IYC is influenced by both social and economic drivers, such as culture, ease of processing and cooking, and limited knowledge of their health implications. Generally, rural households produce crops for sale in distant urban markets rather than for local nourishment.

## 4 Early Childhood Dietary Exposure to Aflatoxins and Fumonisins in Tanzania

Aflatoxins and FBs are secondary metabolites produced by fungi, with aflatoxin B1 (AFB1) and fumonisin (FB1) identified as the most harmful mycotoxins impacting agricultural products, particularly maize (Hove et al. 2016). Aflatoxins are primarily synthesized by the toxigenic fungi *Aspergillus flavus* and *Aspergillus parasiticus* (IARC 1993), while FBs are mainly produced by *Fusarium* species (Guzman and Casteel 1994). The presence of AFs or FBs in food products (whether non‐, semi‐, or fully processed) may indicate contamination that occurs mainly either in the field (by *Fusarium*) during crop development (pre‐harvest) or during storage (by *Aspergillus*). However, *Aspergillus* can infect crops at various stages: pre‐harvest, during harvest, and post‐harvest, which can result in AFs contamination (Gachara et al. 2024). This phenomenon is particularly concerning in humid tropical conditions, where the fungi may colonize crops still in the field and continue to grow after harvest. It is important to note that cereals and nuts are particularly susceptible to contamination by various field and storage fungi and their associated toxins (Ali et al. 2023). Additionally, co‐exposure to these mycotoxins can intensify their toxicological effects.

In Tanzania, AFs and FBs are widely associated with CFs like maize and/or groundnut‐based foods (C. Chen, Mitchell, et al. 2018; Kimanya et al. 2008, Kimanya et al. 2014; Mollay et al. 2022; Shirima et al. 2013). Maize serves as the primary dietary staple in Tanzania. Kimanya et al. (2014) observed that AFs and FBs co‐occurred in 29% of maize flour samples designated for complementary foods. Moreover, these complementary foods primarily comprise maize (Mollay et al. 2021). This dependence on maize‐based complementary foods may expose IYC in Tanzania to both AFs and FBs concurrently, leading to synergistic effects from the toxins. Furthermore, combined exposure to AFs and FBs, or exposure to FBs alone, may exacerbate child stunting (Shirima et al. 2015).

The childhood dietary exposure to AFs and FBs in Tanzania has, in recent times, been revised by Mollay et al. (2020). According to the available reports, the dietary exposure of Tanzanian IYC AFs and FBs occurs at a very young age (< 6 months) (Lombard 2014; Shirima et al. 2013). This is particularly worrying because children are more vulnerable to toxins owing to low body weights, underdeveloped organs, and the inability to detoxify (Lombard 2014), which can subsequently affect their health and development from the associated effects of AFs on the immune system, growth, and body organs (Kimanya et al. 2014; Magoha et al. 2016). This high exposure can possibly be explained by the early introduction of cereal‐based CFs (Khlangwiset et al. 2011; Shirima et al. 2013), which are vulnerable to contamination with mycotoxigenic fungi during growth, pre‐and post‐harvesting, drying, transportation, or storage (Lombard 2014; Shabani et al. 2015). A summary of early childhood dietary exposures to AFs and FBs in Tanzania is provided in Table 1.

**TABLE 1:** Occurrence of aflatoxins and fumonisins in complementary foods and early childhood dietary exposure to the mycotoxins in Tanzania.

Region	Specified area/district	Type of Mycotoxin	Age bracket (months)	Occurrence (μg/kg)	Dietary exposure (ŋg/kgBw/day)	Food consumed	Year of study	References
Dodoma	Dodoma Municipality, Chamwino	Aflatoxins	6–23	0.3–2128.0	0.1–23172.81	Maize, sorghum, millet	NM	Makori et al. (2019)
	Kongwa	Aflatoxin B1	6–23	38.3–271	**	Maize, groundnut	2015	Anitha et al. (2020)
Iringa	Nyabula	Aflatoxins	NM	0.15–31.3	**	Maize	2010–2011	Geary et al. (2016)
	NM	Aflatoxins	6–12	*	15.0‐2537.0 [61.0^b^]	Maize	2016	Kimanya et al. (2021)
Kilimanjaro	Rombo	Fumonisins	6	21–3201	0.003–28.838 [0.48^b^]	Maize	2006	Kimanya et al. (2010)
	Rombo	Aflatoxins	18–24	0.11–386	1–786	Maize	NM	Kimanya et al. (2014)
	Rombo	Fumonisins	18–24	63–2284	0.19–26.37	Maize	NM	Kimanya et al. (2014)
	Kikelelwa	Aflatoxins	NM	0.25–39.7	**	Maize	2010–2011	Geary et al. (2016)
	NM	Aflatoxins	6–12	*	15.0‐6312.0 [105.5^b^]	Maize	2016	Kimanya et al. (2021)
Manyara	Hanang’	Aflatoxins	6–12	3.3^a^	13.4‐14.3 [14^a^]	Maize	NM	Kamala et al. (2017)
	Hanang’	Fumonisins	6–12	193^a^	1.1‐1.24 [1.2^a^]	Maize	NM	Kamala et al. (2017)
Mbeya	Rungwe	Aflatoxins	6–12	4.2^a^	9.2‐9.7 [9.4^a^]	Maize	NM	Kamala et al. (2017)
	Rungwe	Fumonisins	6–12	5267^a^	15‐19 [16.9^a^]	Maize	NM	Kamala et al. (2017)
Morogoro	Morogoro Rural	Aflatoxins	6–24	3.40–78	8.79–549.2 [67.97^a^]	Maize and other grains	NM	Modest (2017)
	Kilosa	Aflatoxins	6–12	124^a^	700–716 [708^a^]	Maize	NM	Kamala et al. (2017)
	Kilosa	Fumonisins	6–12	2808^a^	23‐24 [23.5^a^]	Maize	NM	Kamala et al. (2017)
Tabora	Kigwa	Aflatoxins	NM	0.20–43.65	**	Maize	2010–2011	Geary et al. (2016)
	NM	Aflatoxins	6–12	*	15.0‐10926.0 [242.5^b^]	Maize	2016	Kimanya et al. (2021)
Iringa, Kilimanjaro, Tabora	NM	Aflatoxins	12–22	*	81.7^a^	Maize	2009–2010	Routledge et al. (2014)

*Note:* *Occurrence/contamination was not established, **Exposure was not determined.

Abbreviation: NM, not mentioned.

^a^ Mean.

^b^ Median.

The main CFs in Tanzania mainly include porridge composed of maize, groundnuts, soybeans, sorghum, and rice, which are all vulnerable to aflatoxigenic fungi (Rushunju et al. 2013). These feeding habits may explain the high exposure levels of Tanzanian children to AFs through complementary feeding (Kimanya et al. 2014; Magoha et al. 2016; Shirima et al. 2015). Children fed on maize‐based CFs are especially at a higher risk of exposure to AFs (Shirima et al. 2013). Several studies in the country have revealed a high exposure in IYC to AFs through maize‐based diets (Kimanya et al. 2008, Kimanya et al. 2014; Shirima et al. 2013; Suleiman et al. 2017). Complementary foods are also commonly constituted of groundnuts, which are usually very susceptible to contamination with AFs (Muhimbula and Issa‐Zacharia 2010; Shirima et al. 2015). According to Chang et al. (2013), while peanuts offer nutritional benefits in composite flours, they are also susceptible to contamination with AFs. Previous surveillance in Tanzania revealed that the contamination of peanuts with AFs ranged from 10.3 to 40.3 μg/kg (Abt 2013), which is higher than acceptable levels (Kuhumba et al. 2018; TBS 2014).

A number of IYC in Tanzania are exposed to AFs and FBs beyond the maximum allowable national and international limits as provided by the Joint FAO/WHO Expert Committee on Food Additives (JECFA). For instance, in a study by Kimanya et al. (2009), 10% of young children (> 6 months old) showed intake of FBs above the provisional maximum tolerable daily intake (PMTDI) of 2 μg/kg per body weight from the intake of contaminated maize. The study reported that this percentage would increase to 24% if the maize used to prepare CFs was not sorted before use. Similarly, 32% of children in Rombo district were established to consume maize flours contaminated with AFs (0.11–386 μg/kg), and 30% of the homemade CFs in the district contained AFs above the maximum tolerable limit of 10 μg/kg (Kimanya et al. 2014). Similarly, 56% of the children were exposed to FBs exceeding the PMTDI (Kimanya et al. 2014). In a more recent study, about 73.2% of children 6–24 months old in Morogoro Rural district were reported to consume porridge from flour with detectable levels of AFs (3.40–78 μg/kg) above the tolerable limit of 10 μg/kg and the exposure level to AFs for more than half of them was 8.79–549.2 ng/kg bw/day which surpassed the limit of 0.017 ng/kg bw/day (Modest 2017).

According to Rushunju et al. (2013), 30% of the homemade CFs in Dar es Salaam and Arusha contained AFs above the maximum tolerable limit of 10 μg/kg. In a more recent study by Makori et al. (2019), AFs were identified in 42.5% of homemade complementary maize flours in the range of 0.3–2128 μg/kg. In the same study, the levels of AFs in 30.6% of samples also exceeded the national regulatory limit of 10 μg/kg. Kamala et al. (2017) also established that 50% of all maize‐based food samples meant for human consumption were contaminated with AFs, and 28% of these exceeded the limit of 5 μg/kg.

Some practices can increase or decrease the risk of exposure of infants to AFs and FBs. While modeling the Tanzanian maize consumption data on the occurrence of FBs in sorted and unsorted maize in 2005 and 2006, 26% and 3% of the IYC exceeded the PMTDI of 2 μg/kg put in place by the Joint FAO/WHO Expert Committee on Food Additives (JECFA 2002; Kimanya et al. 2009). In the study, the 50th and 97th percentiles of the consumers in 2005 were exposed to FBs mean levels of 0.47 and 36.99 μg/kg bw/day, respectively. However, a lesser exposure rate of 0.15 and 2.06 μg/kg bw/day occurred in 2006 for the two percentiles, respectively. In the same study, the consumption of unsorted maize by IYC in Rombo district of the Kilimanjaro region resulted in significantly higher exposure to FBs (8.87 μg/kg bw/day) than sorted maize (0.28 μg/kg bw/day). A similar study by Aron et al. (2017) in Bahi district of Dodoma region also reported that some post‐harvest handling practices, for instance, drying on bare grounds and prolonged storage of agricultural produce used in the preparation of CFS are highly associated with contamination by AFs, while processes like winnowing, dehulling, roasting, sorting, and boiling all reduced the quantities of AFs in complementary flours. Nevertheless, it is key to note that although the processing of food can cause the degradation of mycotoxins, mycotoxins can still occur in processed products (Karlovsky et al. 2016).

The IYC are at high risk of AFs' health effects due to their relatively small body weight, low dietary diversity, underdeveloped immune system, and immature metabolic system. Children's exposure to AFs increases with age up to 3 years, with a significant increase during weaning (Gong et al. 2003, 2016). Dietary Aflatoxin exposure risk factors in Tanzania are mainly due to early initiation of complementary food (Magoha et al. 2016) and the use of ingredients susceptible to AFs like maize and groundnuts (Banerjee and Dwivedi 2020; Mollay et al. 2021, 2022). An increase in age is often associated with increased dietary exposure to mycotoxins (Shirima et al. 2013). This is probably because the quantity of food intake increases as children grow and consequently increases the likelihood of being exposed, especially if the diet is contaminated with AFs (Makori et al. 2019). This demonstrates the collective effect of increased consumption of CFs contaminated with AFs during child growth (Kimanya et al. 2021). This has been established in a study by Modest (2017). An investigation by Kimanya et al. (2010) also established that only 46% of the children in Rombo district exceeded the PMTDI concerning exposure to FBs as opposed to 56% in a later investigation in the same district by Kimanya et al. (2014). This could be because the latter focused on older children (18–24‐month‐olds) who probably consumed more food than the younger ones (6‐month‐olds), which formed the study cohort in the former study. As such, the early introduction of children to CFs is one of the factors that contribute to the high exposure of infants to AFs because they are often susceptible to contamination with mycotoxigenic fungi.

The danger of dietary exposure of infants to AFs and FBs is commonly reported in certain areas in Tanzania. According to the 2015–2016 TDHS, Iringa is one of the four regions in Tanzania with the greatest levels of stunting in children younger than 5 years old (NBS and ICF Macro 2011). Most of these foods and ingredients for CFs in these regions are highly prone to contamination with AFs, which can result in acute and chronic dietary exposure. Kongwa district in central Tanzania is one of the areas with very high exposure to AFs and FBs (Anitha et al. 2020). According to Semali et al. (2015), this is among the districts in central Tanzania that receive little rainfall and, therefore, experience poor harvests. The common food crops in this district are groundnuts and maize, which are often contaminated with AFB_1_ during harvest and even higher contamination levels during storage owing to poor post‐harvest practices (Turner 2013). The occurrence of AFB_1_ in samples of maize from different agro‐ecologies of Tanzania, such as Iringa, Tabora, and Kilimanjaro, has been established (Kimanya et al. 2008). This implies that consumers in such agro‐ecologies may be highly exposed to AFs.

## 5 Child Stunting in Tanzania as Linked to Dietary Exposure to Aflatoxins and Fumonisins

Stunting is an indicator of chronic undernutrition, which occurs when a child has height for age *z*‐scores (HAZ) below −2 standard deviations of the median height‐for‐age of a reference population according to the WHO Multicentre Growth Reference Study Group (2006). Although several factors contribute to child growth impairment, there is a budding focus on comprehending the role of dietary toxins in fueling the risk. Evidence linking the exposure of children to FBs and stunting, especially in regions where young children consume maize‐based CFs, is steadily increasing. Nevertheless, relatively few studies have been conducted in Tanzania on the connection between exposure to FBs and child growth. The first such study established the exposure of 215 Tanzanian 12 month infants to FBs by measuring the levels of FBs in maize flour and then approximating the infants' consumption of maize based on their mothers' dietary recalls (Kimanya et al. 2010). The study established that the growth of infants in the same community was inversely associated with exposure to FBs, and infants exposed to FBs exceeding JECFA's PMTDI of 2 μg/kg bw/day were considerably smaller and lighter compared to those whose intakes were below the JECFA limit.

Chronic exposure to AFs and FBs is associated with child stunting (Anitha et al. 2020; Chen, Mitchell, et al. 2018; Gong et al. 2003; Khlangwiset et al. 2011; Kimanya et al. 2010; Shirima et al. 2015). Co‐exposure of AFs with FBs, which has also been reported in Tanzania, can also intensify the impact of stunting compared to the effect of single toxin exposure (Routledge et al. 2014). A longitudinal study on the exposure of Tanzanian IYC to AFs and FBs as a contributing factor to early childhood growth impairment was conducted by Shirima et al. (2015). The study established a negative relationship between growth and exposure of children to FBs and AFs based on urinary biomarker levels, demonstrating a possible link between exposure to FBs and child growth impairment. While the co‐exposure to the two mycotoxins was significantly associated with impaired growth, the independent exposure to AFs, in contrast, did not significantly impact child growth. This means that exposure to FBs could be a significant risk factor in child stunting (Chen, Mitchell, et al. 2018; Shirima et al. 2015). An association between stunting in these children and their exposure to AFs was also insignificant (Shirima et al. 2015). In a separate study by Makori et al. (2019), the consumption of composite cereals containing groundnuts was also found to be significantly associated with stunted growth (*p* = 0.023) and the odds of stunted growth for children who were fed on composite cereals flours with groundnuts was almost 20 times higher than those fed on single cereal flours. In the same study, a multivariate regression analysis association between stunting and dietary exposure to AFB1 was found to be significant (*p* = 0.05).

Elsewhere, feeding IYC with unsafe complementary foods and relying on maize and groundnut‐based products, which may be contaminated with AFBs and FBs in Tanzania, can considerably contribute to stunted growth in children (Anitha et al. 2020; Mollay et al. 2021; Shirima et al. 2015). Although some surveys have established a non‐significant link between exposure to AFs and the growth of infants in Tanzania, for instance, in Haydom (Mbulu district) (Chen, Mitchell, et al. 2018; Chen, Gong, et al. 2018), Kongwa district (Anitha et al. 2020), and Rombo district (Magoha et al. 2016). The study conducted by Chen, Gong, et al. (2018) was at a greater occurrence rate of 75%, compared to that by Shirima et al. (2015). This hence infers a probable dose‐dependent effect of FBs on child stunting in the country. In another recent study in Morogoro Rural district, the dietary exposure of infants (6–24 months) to AFs through the consumption of contaminated CFs was also established to be significantly associated with stunting (*p* = 0.042) (Modest 2017). In Morogoro, which is listed among the most affected regions in Tanzania in terms of child stunting (TFNC 2015), the early introduction of CFs is a common practice, especially in low‐income households (Muhimbula and Issa‐Zacharia 2010). Unfortunately, the main CFs in this region constitute maize, groundnuts, soybean, sorghum, and rice, which are vulnerable to aflatoxin‐producing fungi (Modest 2017; Rushunju et al. 2013), which could be linked to the reportedly high rates of stunting.

Like other parts of the world, strong evidence still points to the link between early childhood exposure to AFS and FBs and impaired child growth in Tanzania. Like other African countries, the mean concentration of AFs was higher in the albumin of stunted children in Tanzania as opposed to non‐stunted ones in a study that sought to compare the prevalence of aflatoxin‐attributable stunting in children under five in developing countries on the continent (Rasheed et al. 2021). Apart from the dietary exposure of infants to AFs and FBs through CFs, exposure during pregnancy is linked to poor birth outcomes and linear growth retardation in infancy (Hassen et al. 2024; Turner et al. 2007), However, prospective cohort research conducted in rural Malawi indicated that while maternal AF exposure did not correlate with stunted infant growth, child AF exposure did. Furthermore, a sustained reduction in linear growth was associated with exposure at 18 months (Matchado et al. 2023). Furthermore, a substantial inverse link between the exposure of Tanzanian 6‐\month old infants to aflatoxin M1 (AFM1—a metabolite of AFB1) and growth impairment was also previously established by Magoha et al. (2014). Therefore, this avenue of exposure must not be overlooked. The current review revealed the presence of AFs and FBs, either separately or together, in maize and maize‐based foods, with varying levels depending on the season and geographical location of the research area in Tanzania. From the review, several studies indicate that the consumption of maize‐groundnut‐based foods is associated with a risk of exposure to AFs and FBs. Research in Tanzania has shown that the impact of AFs and FBs on a child's growth is dependent on the child's age and level of toxin exposure, as evident in longitudinal studies (G. Chen, Gong, et al. 2018; Shirima et al. 2015). Furthermore, exposure to FBs alone or in combination with AFs may contribute to child stunting (Shirima et al. 2015). However, the findings regarding the association between AFs and child stunting have been inconclusive. Shirima et al. (2015) found no association between child stunting at 24 months and exposure to relatively low levels of AFs. On the other hand, Shirima et al. did not find a significant association between exposure to AFs, measured by AF‐albumin adducts, and stunted growth in children. In contrast, Rasheed et al. (2021) suggested that mitigating exposure to AFs in areas with high levels could potentially save up to 50% of Disability‐Adjusted Life Years (DALYs) associated with stunting. Additionally, an intervention incorporating diversified food resulted in a significant reduction in AFs exposure, with average aflatoxin M1 levels in urine samples decreasing by 64% in the intervention group, whereas the control group had just an 11% reduction (Anitha et al. 2020). Therefore, biomarker‐based prospective studies in Tanzania have found a link between AFs, FBs, chronic exposure, and child stunting. Furthermore, dietary exposure to AFs and FBs has been associated with impaired growth in children, primarily due to the consumption of contaminated maize and groundnut based complementary foods. However, it is crucial to conduct further research to elucidate the causal relationship between AFs, FBs and child stunting. There is a pressing need for additional biomarker studies, such as plasma aflatoxin‐albumin adducts (AF‐alb) and urinary fumonisin B_1_ (UFB_1_), integrated with randomized controlled interventions, nutritional education, dietary assessments, and growth monitoring to investigate the causality of dietary exposure to AFs and FBs and its impact on child stunting across various agroecological zones in Tanzania.

## 6 Mechanisms of Growth Impairment

The mechanisms by which AFs and FBs impact child growth are not yet fully known. According to Smith et al. (2012), there are two probable ways by which these toxins cause child stunting: systemic immune activation and the induction of environmental enteric dysfunction. This results in the interruption of the insulin‐like growth factor 1 (IGF1), whose main function is to stimulate the growth‐promoting effects of growth hormone (Woods et al. 1996). The AFs can also induce stunting in children through an immunosuppressive effect, which in most cases heightens the vulnerability of infants to infection, reduces nutrient absorption, and the loss of appetite (Gong et al. 2008). Besides, AFs may hamper child growth by facilitating intestinal damage through protein synthesis inhibition (Smith et al. 2012).

According to Mupunga et al. (2017), AFs can also interfere with intestinal integrity, which leads to the malabsorption of nutrients, micronutrient deficiencies, impaired immune function, and vulnerability to gut infections, which together may cause impaired growth and malnutrition. Aflatoxins may result in poor nutrient retention and prolonged intestinal infections by mediating gut inflammatory responses. Besides being potent liver toxins, AFs may damage liver cells and disturb nutritional homeostasis during regeneration, thus contributing to growth impairment (Luyendyk et al. 2003). Another possible mechanism of growth impairment involves the inhibition of protein synthesis due to the binding of AFs with nucleic acids and proteins, and therefore causing interference with enzymes and substrates that are required for processes involved in protein synthesis (Bbosa et al. 2013).

Unlike AFs, FBs can impair child growth through the inhibition of ceramide synthase, an important enzyme in the biosynthesis of sphingolipids, thus disrupting sphingolipid metabolism (Yazar and Omurtag 2008). This concurs with Semba et al. (2016) that the significant reduction of serum concentrations of sphingomyelins occurred in stunted Malawian children from Malawi compared to non‐stunted ones. Sphingomyelin is a dominant sphingolipid in mammalian cells with a key function in forming lateral structures in membranes for Toll‐like, scavenger, and insulin receptors (Slotte 2013). The impact of FBs on intestinal barrier function through the modification of the sphingoid base‐1 phosphate signaling pathway has also been established (Bulder et al. 2012). Riley et al. (2015) also established that the levels of urinary FB1 and sphinganine 1‐phosphate/sphingosine 1‐phosphate ratio in blood were positively correlated, a report consistent with the inhibition of ceramide synthase in humans by FBs. Besides the inhibition of ceramide synthase, FBs may also elicit inflammatory responses that damage the gut barrier function (Masching et al. 2016).

Co‐exposure of AFs and FBs may have far more reaching consequences on child growth due to combined effects, but this is still unclear. According to Chen, Mitchell, et al. (2018), co‐exposure of the two mycotoxins can facilitate intestinal damage; thus, malabsorption of nutrients and chronic systemic immune activation that ultimately lead to growth retardation in the long run. This requires further investigation because health risks and growth impairment may be complicated by co‐exposure (Strosnider et al. 2006). Aflatoxin is associated with stunted growth in children because it damages the intestinal lining, thus reducing the absorption of essential nutrients such as proteins, vitamins, and minerals. This impairs the body's ability to utilize these nutrients for growth and development (Gong et al. 2008). Furthermore, studies have reported the association between prolonged AFB1 exposure in children with liver damage (Khlangwiset et al. 2011), immune suppression due to its immunosuppressive effects (Tesfamariam et al. 2020), and endocrine system disruption (Amir et al. 2022). All those interferences for normal function in a child's body can lead to impairment of growth and development and result in stunted growth. A systematic review and meta‐analysis study performed by Nejad et al. (2023) to investigate the association of AFB1 and growth failure in children revealed a negative association between higher AFB1 exposure. In the study of 17 studies with a total sample size of 5633 participants, a pooled analysis of all available effect sizes found an inverse association between exposure to AFB1 and growth failure measurements (height, HAZ, and stunting). Therefore, although the evidence remains unclear, AFs, whether present alone or in combination with FBs, may collectively reduce nutrient utilization and significantly contribute to child growth impairments.

## 7 Past, Present, and Future Interventions

Child stunting remains a global public health challenge, and reducing it is among the six global nutrition goals advanced by the World Health Assembly (de Onis et al. 2013; WHO 2015). The high rates of stunting in Tanzania reflect the presence of severe undernutrition and highlight an urgent requirement to prioritize appropriate interventions (Mbwana et al. 2017). At the moment, different interventions have been put in place to mitigate stunting, with a lot of stress on exclusively breastfeeding children for 6 months and proper complementary feeding (Musheiguza et al. 2021). ‘Meet nutrition needs in Iringa, Njombe, and Mbeya regions of Tanzania’ is an initiative of the Tanzanian government, UNICEF, and Concern Worldwide, an international non‐governmental organization, to lessen the prevalence of stunting in the country by 10% within 5 years through interventions aiming at mothers and children and strengthening capacities of local authorities (Altare et al. 2016).

Tanzania has been fighting to reduce stunting, with a prevalence of 4% reduction from 2015 to 2022. The prevalence of stunting was expected to reduce to 28% by 2021 as required by Sustainable Development Goal (SDG) 2.2.1. According to WHO (2018), by the year 2025, the worldwide stunting rate has to be lowered by 40%. Failure to do so could put children at risk of the long‐term effects of stunting and may prevent them from reaching their full growth potential (Black et al. 2013). Stunting is alterable in the first 1000 days of a child's life, after which it becomes irreversible (Black et al. 2013; Kejo et al. 2018), and significant efforts and interventions that promote child health during this time may address the challenge (Black et al. 2013).

The association between early childhood exposure to AFs and FBs and child stunting in Tanzania is now clearer. Since the exposure of children to AFs increases markedly through complementary feeding, reducing the levels of AFs in CFs is crucial. Interventions to lower the levels of AFs and FBs in crops can be grouped into pre‐and post‐harvest methods. During the preharvest stage in the field, they may use Aflasafe, which is a biocontrol technology to prevent aflatoxin‐producing fungi from contaminating maize in the field. Once applied, Aflasafe contains harmless, atoxigenic (non‐toxin‐producing) strains of *A. flavus* . These strains outcompete the toxic ones in the soil and on the crop. Aflasafe strains normally dominate the environment, reducing the chance of aflatoxin‐producing fungi infecting the maize. Post‐harvest practices, like some traditional/cultural methods of handling maize before consumption, have proven to be efficient in decreasing FBs. Proper processing of food ingredients by dehulling, sorting, and washing can also reduce concentrations of FBs in maize (Kamala et al. 2017; Maseta et al. 2016). These low‐cost processes target contaminated grain parts and infected kernels that may harbor fungal contamination. Mechanical dehulling (Fandohan et al. 2005) and traditional dehulling (Kimanya et al. 2008) can effectively reduce fumonisin contamination levels in maize. Sorting can eliminate visibly damaged or moldy kernels, which are more likely to contain higher levels of AFs and FBs (Kimanya et al. 2008). Furthermore, combining size and density sorting maximizes fumonisin removal by excluding both small or light kernels and visibly damaged ones (Stafstrom 2023). Additionally, when sorting is paired with washing, it can reduce the surface contamination that may carry fungal toxins, such as AFs and FBs to the surfaces of grains. The mycotoxin content in grains is partially reduced through cleaning and milling processes. Higher levels of mycotoxins are typically found in the outer layers of the grain, which are reduced during milling or dehulling (Cheli et al. 2013; Tibola et al. 2016). These practices are appropriate to rural settings and have also proven to be effective intervention strategies in reducing FBs in other parts of Africa (Fandohan et al. 2005; Matumba et al. 2015; Van der Westhuizen et al. 2010). Furthermore, these interventions have recently been demonstrated to decrease the diet‐related exposure of children to AFs, FBs and undernutrition among Tanzanian children (Kamala et al. 2018).

Maternal education can significantly reduce the risks of child stunting and might be a basic dynamic in proper infant feeding practices (Chirande et al. 2015; Meena et al. 2015). The education of mothers should also entail the campaign for dietary diversification using alternative foods like wheat, finger millet, and legumes as CFs to lower the risk of mycotoxin contamination and exposure to infants (Gheysens 2015). A very recent study by Anitha et al. (2020) provides quantitative evidence on the positive impacts of children's nutritional outcomes in four rural areas in the central Tanzanian district of Kongwa, following an intensive 21‐day maternal education in terms of food safety, dietary diversification, and hygiene interventions. High exposure of infants to mycotoxins can either be due to the elevated consumption levels of averagely contaminated foods or the moderate intake of highly contaminated foods (Kamala et al. 2017). Therefore, one possible way of lowering the threat of dietary exposure to mycotoxins in the country is by limiting the consumption of commonly contaminated foods as well as the mycotoxin levels in these foods. Therefore, advocacy for diet diversification in consideration of locally available foods that are less susceptible to contamination by mycotoxins, alongside the aforementioned pre‐and postharvest handling methods, is urgently required to minimize the exposure of Tanzanian children to mycotoxins in maize and other CFs (Kamala et al. 2017).

There seems to be substantial regional variation concerning stunting, but the prevalence is relatively well documented at the regional levels as opposed to the districts. This is probably because the TDHS only avails national and regional data on the nutritional status of a child. Similarly, the TDHS does not evaluate the factors that affect child nutritional status at the regional and district levels. This is likely to affect the form of intervention introduced in specific districts to overcome child undernutrition. Specific interventions that target local factors that influence stunting in different regions may substantially reduce child stunting by 2026. Intervention packages like nutritional, health, and Water, Sanitation, and Hygiene interventions to decrease childhood stunting should also encompass the reduction of exposure of infants to AFs and FBs, with a priority focus on stunted children and those affected not only by stunting but also underweight (Rasheed et al. 2021).

## 8 Concluding Remarks

Child stunting is the most prevalent type of childhood chronic undernutrition, and its allied factors are diverse and interrelated. The current review discusses child stunting and nutrition and strives to draw a link between early childhood dietary exposure to AFs and FBs and child stunting in Tanzania. Chronic low exposure to AFs affects child growth. Extra efforts are therefore necessary to reinforce nutrition interventions that can also improve the quality of CFs and child nutrition. As such, interventions aimed at reducing AF and FB exposure are recommended, including a focus on dietary diversification, post‐harvest processing, and maternal education regarding the feeding of IYC with safe and nutritious foods that are locally available, as well as optimal complementary feeding practices. The locally available foods and their products are not only affordable but also accessible within their environment, which makes complementary feeding more economically viable and ensures that children receive a diverse and nutritious diet most of the time. Furthermore, maternal education will empower mothers to make informed choices about nutritious and safe foods, as well as appropriate food production and preparation practices. To address child stunting in Tanzania, these efforts can be complemented by biomarker development, intervention trials, child growth monitoring, and the evaluation of food and nutrition policies. The general intent is to induce more policy actions for the mitigation of the problem in the country. This evidence is crucial because, in the absence of an aflatoxicosis outbreak, policymakers may not comprehend the magnitude of the health impacts of chronic exposure of IYC to AFs since the toxins are invisible and stunting manifests long after exposure. This review can be crucial for developing and adopting appropriate interventions that can contribute to the further reduction of child stunting in Tanzania.

## Author Contributions

**Clara Mollay:** conceptualization, literature review, validation, and writing – including the original draft, review, and editing.

## Funding

The author has nothing to report.

## Conflicts of Interest

The author declares no conflicts of interest.

## Data Availability

Data sharing is not applicable to this article, as it is solely a review article.
